# Profiling single nucleotide polymorphisms (SNPs) across intracellular folate metabolic pathway in healthy Indians

**Published:** 2011-03

**Authors:** Yogita Ghodke, Arvind Chopra, Pooja Shintre, Amrutesh Puranik, Kalpana Joshi, Bhushan Patwardhan

**Affiliations:** *Interdisciplinary School of Health Sciences (ISHS), University of Pune, Pune, India*; * *Centre for Rheumatic Diseases (CRD), Sinhgad College of Engineering, Pune, India*; ** *Department of biotechnology, Sinhgad College of Engineering, Pune, India*

**Keywords:** Folate metabolism, Indians, methotrexate (MTX), pharmacogenomics, single nucleotide polymorphism (SNP)

## Abstract

**Background & objectives:**

Many pharmacologically-relevant polymorphisms show variability among different populations. Though limited, data from Caucasian subjects have reported several single nucleotide polymorphism (SNPs) in folate biosynthetic pathway. These SNPs may be subjected to racial and ethnic differences. We carried out a study to determine the allelic frequencies of these SNPs in an Indian ethnic population.

**Methods:**

Whole blood samples were withdrawn from 144 unrelated healthy subjects from west India. DNA was extracted and genotyping was performed using PCR-RFLP and Real-time Taqman allelic discrimination for 12 polymorphisms in 9 genes of folate-methotrexate (MTX) metabolism.

**Results:**

Allele frequencies were obtained for *MTHFR* 677T (10%) and 1298 C (30%), *TS 3UTR* 0bp (46%), *MDR1* 3435T and 1236T (62%), *RFC1* 80A (57%), *GGH* 401T (61%), *MS* 2756G (34%), *ATIC* 347G (52%) and *SHMT1* 1420T (80%) in healthy subjects (frequency of underlined SNPs were different from published study data of European and African populations).

**Interpretation & conclusions:**

The current study describes the distribution of folate biosynthetic pathway SNPs in healthy Indians and validates the previous finding of differences due to race and ethnicity. Our results pave way to study the pharmacogenomics of MTX in the Indian population.

Folate, a water soluble B vitamin, plays a key role in one-carbon metabolism. It is an essential cofactor for *de novo* biosynthesis of purine and thymidine nucleotide^1^,^2^ (Fig.) with special reference to methylation reactions and epigenetic influences (DNA, chromosomes and mutations)^3^. Folate deficiency causes anaemia and is considered to be of aetiopathogenetic importance in several cardiovascular diseases, neural tube defects and other congenital defects, adverse pregnancy outcomes, neuropsychiatric and cognitive disorders, and cancer^4^,^5^. Folate antagonists like methotrexate (MTX) and 5 fluorouracil target folate metabolism. Folate analogue are also widely used to treat cancer, autoimmune diseases, psoriasis, and infections.

**Fig:**
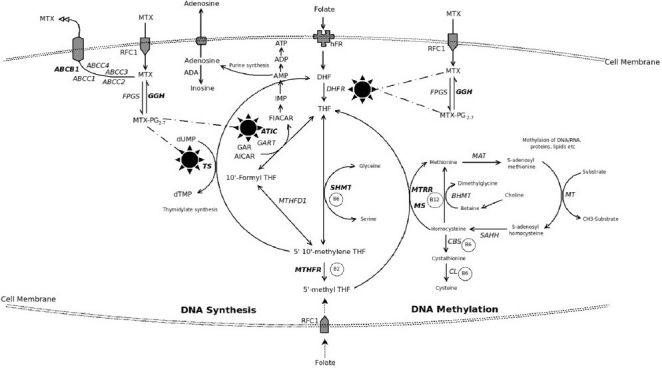
Intracellular pathway of folate metabolism with highlighted critical pathways of methotrexate (MTX) entry, effect and efflux. Folate enters cell as methyl tetrahydrofolate (MTHF) through a folate carrier receptor system and is converted into tetrahydrofolate (THF). MTHF is the co-substrate for production of methionine (from homocysteine) and polyamines. Inside cell, folates as polyglutamates (PG) create an inter-convertible pool (ICP) which promotes several reactions with 1-carbon fragments wherein methyl and formyl groups are donated (primarily by serine to glycine) and this leads to various nucleic acid/nucleotide biosynthesis. All 1-carbon transfer reactions regenerate THF. The exception to the latter is production of dihdrofolate (DHF) from folate substrate while converting deoxyuridylate (dUMP) to dTMP. An important enzyme dihydrofolate reductase (DHFR) converts DHF to THF which is the essential active form of folate in the ICP. Several drugs, including MTX block DHFR to produce a relative state of intracellular folate deficiency while the tissues are rich in folates. MTX is a competitive inhibitor of DHFR and enters the cell through reduced folate carrier (RFC) active transport system. MTX forms PG intracellularly by the enzyme folylpolygluatamate synthase (FPGS); another enzyme gamma glutamyl hydrolase (GGH) reverses this process to cause MTX efflux from the cell. The PG forms retain MTX inside the cell as MTXPG and is responsible for its several effects besides inhibiting DHFR, (*i*) it inhibits thymidylate synthetase (TS) which converts deoxyuridylate (dUMP) to dTMP in the de novo pyrimidine pathway, (*ii*) though not a direct target of MTX, methylenetetrahydrofoate reductase (MTHFR) to an important enzyme in the folic acid pathway which is influenced by the MTX effects on the intracellular folate pool, (*iii*) it affects purine synthesis pathways by inhibiting 5-aminoimidazole-4-carboxamide ribonucleotide formyltranferase (ATIC) to cause intracellular accumulation of 5-aminoimidazole-4-carboxamide ribonucleotide (AICAR). AICAR inhibits adenosine deaminase (ADA) and AMP deaminase to cause intracellular accumulation of adenosine and related nucleotides which then get dephosphorylated to finally result in increased extracellular adenosine. Adenosine is a potent anti-inflammatory agent. Methionine synthase (MS) converts homocysteine (if excess can confer cardiac risk) to methionine required for several cellular DNA/RNA metabolism pathways. Serine hydroxymethyltransferase1 (SHMT1) operates on the DHF-THF pathway. ATP-binding cassette (ABC) family (7 distinct families A-G) of transporter proteins are responsible for MTX efflux from the cell. Multidrug resistant protein1 (MDR1), an active transporter system, is related to the ABC system in expelling several organic anions including MTX from the cell.

Recently, several studies have described SNPs of the genes involved in folate metabolism^6^ and their role in related diseases^7^–^10^. Though inadequate, data also suggest that these SNPs may influence therapeutic outcome by playing a critical role in the metabolism of drugs targeting folate biosynthetic pathway^6^,^11^. Importantly, racial and ethnic differences in the occurrence of SNPs have been proposed^12^–^14^.

The data on the distribution of SNPs (folate pathway) in the Indian population are sparse. Any study on the frequencies of SNPs in disease must be preceded by their distribution in the healthy population. The present study aims to determine the allelic frequency of SNPs across intracellular folate metabolic pathway in healthy Indian subjects. Allele frequencies were also compared with previously reported frequencies by others to address the racial and inter-ethnic differences.

## Material and Methods

### 

#### Study population:

One hundred and forty four unrelated healthy subjects of either sex were enrolled in the study from the outpatient rheumatology referral service of Centre for Rheumatic diseases, Pune (west India) from April 2007- January 2008. There were 70 males and 74 females with mean age (SD) of 23.22 (5.3) years. The study protocol was approved by the Ethics Committee of Centre for Rheumatic Diseases, Pune. Subjects willing to participate provided consent as per the guidelines from the Institutional ethics Committee.

#### Genotype analysis:

Post-consent, peripheral blood sample (4-5 ml) was drawn from each healthy subject, and genomic DNA was extracted using Miller’s protocol^15^. A total of 12 polymorphisms in 9 genes of MTX metabolism (including transporters) were studied. The genes analyzed were *MTHFR: Methylenetetrahydrofoate reductase; TS: Thymidylate synthase; RFC1: Reduce folate carrier1; MS: Methionine synthase; SHMT1: Serine hydroxymethyltransferase1; MDR1: Multidrug resistant protein1; GGH: γ glutamyl hydrolase; ATIC: Aminoimidazol carboxamide ribonucleotide transformylase; MTRR: Methionine synthase reductase*. Genotyping was performed using PCR-RFLP technique for *MTHFR* A1298C (rs1801131) and C677T (rs1801133), *TS 5’UTR* repeat and *3’UTR* deletion, *RFC1*G80A (rs1051266), *MS* A2756G (rs1805087), *MDR1*C3435T (rs1045642) and C1236T (rs1128503), *GGH* C401T (rs3758149), *MTRR* A66G (rs1801394) polymorphisms (oligonucleotides-Integrated Biotechnologies, restriction endonucleases-New England Biolabs)^16^–^18^. Real-time Taqman allelic discrimination assay (Applied Biosystems, CA, USA) was used for genotyping *ATIC* C347G (rs2372536), *SHMT1*C1420T (rs17829445) polymorphisms^19^. After restriction digestion, digested products were visualized on 2 per cent agarose gel except for 5’UTR repeats of *TS* which were directly visualized after the PCR. Real-time Taqman allelic discrimination assays were performed according to protocols provided by the manufacturer (Applied Biosystems, CA, USA). Samples containing mutants were reanalyzed to ensure the accuracy of the method. There was 100 per cent reproducibility.

#### Statistical analysis:

Statistical analysis was performed using the Graph Pad Prism statistical software (San Diego CA. USA). Allele frequencies were determined for 12 polymorphisms in nine genes in the folate-MTX metabolic pathway in 144 healthy subjects. The frequency of each allele in the study population is given in the Table I. Differences in allele frequencies between healthy subjects and other ethnic groups were measured by Fisher exact test. *P*< 0.05 was considered statistically significant. The observed genotype frequencies of polymorphisms studied were compared with expected frequencies according to Hardy-Weinberg equilibrium (HWE) using χ^2^ tests.

**Table I:** The allele frequency of SNP across intracellular folate metabolic pathway in Indian population in the current study and comparison with others

Polymorphism	Present study healthy subjects n=144	European^14^ healthy subjects n=95	African^14^ healthy subjects n=95	Indian^20^ healthy subjects n=77

*MTHFR* C677T				
C allele	0.90	0.68	0.96	NA
T allele	0.10	0.32**	0.04*	
*MTHFR* A1298C				
A allele	0.70	0.72	0.87	NA
C allele	0.30	0.29	0.13**	
*TS* 5UTR				
2R allele	0.36	NA	NA	NA
3R allele	0.63			
*TS* 3UTR				
6bp allele	0.52	0.73	0.44	NA
0bp allele	0.46	0.27**	0.56	
*MDR1* C3435T				
C allele	0.38	0.46	0.90	0.35
T allele	0.62	0.54	0.10**	0.65
*MDR1* C1236T				
C allele	0.38	0.54	0.86	0.28
T allele	0.62	0.46	0.14**	0.72*
*RFC1* G80A				
G allele	0.43	NA	NA	0.72
A allele	0.57			0.28**
GGH-401				
C allele	0.38	NA	NA	0.75
T allele	0.61			0.25**
MS A2756G				
A allele	0.66	NA	NA	NA
G allele	0.34			
MTRR A66G				
A allele	0.50	NA	NA	NA
G allele	0.50			
ATIC C347G				
C allele	0.48	NA	NA	NA
G allele	0.52			
SHMT1 C1420T				
C allele	0.20	NA	NA	NA
T allele	0.80			

P^*^<0.05

** <0.001 compared to present study; NA, Data not available

## Results and Discussion

We examined allele frequencies for 12 polymorphisms in folate and MTX metabolism among healthy subjects and compared them with the allele distribution in other ethnic groups (Table I). Allele frequencies obtained for the present study were *MTHFR* 677T (10%) and 1298 C (30%), *TS 3UTR* 0bp (46%), MDR1 3435T and 1236T (62%), *RFC1* 80A (57%), *GGH* 401T (61%), *MS* 2756G (34%), *ATIC* 347G (52%) and *SHMT1* 1420T (80%). The complete genotype distribution for healthy subjects is represented in Table II. Genotype frequencies for all 12 SNPs were in HWE for healthy subjects.

Healthy subjects from our study were compared with healthy subjects from European, African and Indian population (Table I). *MTHFR* 677T variant allele frequency in European population (32%, *P*<0.001) was higher than Indian healthy subjects (10%) while TS 3 UTR 0bp (deletion) polymorphism was lower in European (27%, *P*<0. 001) than Indian (46%). There was no difference in distribution of *MTHFR* 1298C, *MDR1* 1236T and *MDR1* 3435T variant allele frequencies between Indian and European healthy subjects. The occurrence of *MTHFR* 677T (4%, *P*<0.001), *MTHFR* 1298C (13%, *P*<0.001), *MDR1* 3435T (10%, *P*<0.001) and *MDR1* 1236T (14%, *P*<0.001) variant alleles was significantly lower in Africans as against Indian healthy subjects.

**Table II:** Genotype distribution of 12 SNPs in folate metabolism among healthy subjects

Polymorphism	Healthy subjects n=144		
	Observed frequency	Expected frequency by Hardy-Weinberg law	*P* value

MTHFR C677T			
CC	0.81	0.80	
CT	0.17	0.19	
TT	0.02	0.01	0.79
MTHFR A1298C			
AA	0.48	0.51	
AC	0.46	0.41	
CC	0.06	0.08	0.72
TS5UTR^*^			
2R/2R	0.19	0.13	
2R/3R	0.34	0.45	
3R/3R	0.46	0.40	0.22
TS 3UTR			
0bp/0bp	0.23	0.29	
6bp/0bp	0.47	0.50	
6bp/6bp	0.31	0.21	0.25
MDR1 C3435T			
CC	0.14	0.15	
CT	0.49	0.47	
TT	0.37	0.38	0.95
MDR1 C1236T			
CC	0.13	0.15	
CT	0.50	0.47	
TT	0.37	0.38	0.88
RFC1 G80A			
GG	0.27	0.19	
GA	0.33	0.49	
AA	0.40	0.32	0.07
MSA2756G			
AA	0.41	0.43	
AG	0.51	0.45	
GG	0.08	0.12	0.54
MTRR A66G			
AA	0.26	0.25	
AG	0.49	0.50	
GG	0.25	0.25	0.98
GGH-401			
CC	0.14	0.15	
CT	0.49	0.47	
TT	0.37	0.38	0.95
ATIC C347G			
CC	0.23	0.23	
CG	0.50	0.50	
GG	0.27	0.27	1.00
SHMT1 C1420T			
CC	0.02	0.04	
CT	0.36	0.32	
TT	0.62	0.64	0.62

Comparison of our healthy subjects with Indian study from north India reveals that there was significant difference in the occurrence of *GGH* 401T, *RFC1* 80A and *MDR1* 1236T variant alleles. The occurrence of *GGH* 401T (61%) andRFC1 80A (57%) in our healthy subjects was higher than north Indian subjects 25 and 28 per cent (*P*<0.0001) respectively while *MDR1* 1236T was higher in north Indians (72%) than our healthy subjects (62%, *P*<0.05). Thus the current report supports the previous findings that the allele or haplotype frequencies of several important polymorphisms in folate pathway vary with race^12^–^14^.

The allele frequencies of *MTHFR* 1298C and 677T, *TYMS* 3’ UTR deletion and *MDR1* 3435T and 1236T in healthy subjects are different in Indian subjects as compared to Europeans and Africans^12^. The latter conclusion is limited by the fact that we could only find data on five polymorphisms in reports of European and African healthy population. We have also compared our data with north Indian population^20^. There are differences in the occurrence of *GGH* 401T, *RFC1* 80A and *MDR1* 1236T variant alleles within Indian population. This intra-ethnic difference can be because Indian population is a conglomeration of multiple culture and evolutionary histories. The evolutionary antiquity of Indian ethnic groups and subsequent migration from central Asia, west Asia and southern China has resulted in a rich tapestry of socio-cultural, linguistic and biological diversity^21^.

SNPs have been reported *per se* to impair folate-mediated one-carbon metabolic pathways and contribute to increased risk of several disorders of folate deficiency^22^–^24^. Folate antagonist MTX is among the best-tolerated disease-modifying antirheumatic drugs (DMARDs) used in the treatment of RA, but is confounded by unpredicted interpatient variability in clinical response and toxicity^6^,^25^. To unravel the probable associations among variations in drug pathway alleles and MTX response in Indian rheumatoid arthritis (RA) patients, it is essential to first explore the relationship between the genes coding for folate metabolic pathway and ethnicity. The results of the current study are a step forward in that direction.

To our knowledge this is the first report on 12 polymorphisms in 9 genes of folate metabolic pathway in Indian population. We have not analyzed polymorphisms in *folypolyglutamate synthase (FPGS)* and *dihydrofolate reductase (DHFR)*. We report ethnic differences in the SNPs in genes coding folate biosynthetic metabolic intracellular pathway. It may not be appropriate to extrapolate the findings of genetic associations influencing folate antagonist treatment response in subjects belonging to Caucasian and African ethnicity to the Indian population. Thus knowledge of allelic frequency distribution within a population can be useful in optimizing doses for therapeutic efficacy, identifying potential risk groups for adverse drug reactions and explaining therapeutic failures.
